# Simultaneous Determination of Nine Phthalates in Vegetable Oil by Atmospheric Pressure Gas Chromatography with Tandem Mass Spectrometry (APGC-MS/MS)

**DOI:** 10.3390/toxics11030200

**Published:** 2023-02-21

**Authors:** Yongjun Xiao, Wen Yee Wong, Li Yan Chan, Chee Keat Yong, Kosuke Abe, Peter Hancock, Simon Hird

**Affiliations:** 1 International Food & Water Research Centre, Waters Pacific Pte Ltd., 1 Science Park Road #01-10, The Capricorn, Singapore Science Park II, Singapore 117528, Singapore; 2 Nisshin Global Research Center Sdn. Bhd., Lot. 1, Lebuh Sultan Hishamudin 2, Kawasan 20, Bandar Sultan Suleiman, Port Klang 42009, Selangor, Malaysia; 3 Waters Corporation, Stamford Avenue, Altrincham Road, Wilmslow, Cheshire SK9 4AX, UK

**Keywords:** atmospheric pressure gas chromatography (APGC), GC–MS/MS, phthalates

## Abstract

Although the use of phthalates has been restricted worldwide, they remain an issue due to health concerns. Diet is one of the most important exposure pathways for humans and due to their solubility in oil, phthalates are commonly found in edible oil and food high in fat. Gas chromatography–mass spectrometry (GC-MS) using electron ionization (EI) has been commonly used for the analysis of the phthalates in foodstuffs, including edible oil. However, this method suffers from issues with sensitivity and selectivity, as most phthalates are fragmented to generate a common phthalic anhydride fragment ion at *m/z* 149. The molecular ion cannot be observed due to strong fragmentation in EI. In contrast, atmospheric pressure gas chromatography (APGC) is a soft ionization technique with less fragmentation, whereby the molecular ion can be used as the precursor ion for multiple reaction monitoring (MRM). In this study, a simple and quick method for the determination of phthalates in vegetable oil using APGC-MS/MS was developed, and performance was assessed. The method was based on dilution of the oil in solvent and direct injection without the need for further cleanup. The established method was evaluated for linearity, recovery, precision, method detection limit (MDL), and method quantitation limit (MQL). The obtained MQL in vegetable oil was in the range of 0.015–0.058 mg/kg, despite limiting the injection volume to 1 µL, which is suitable for investigating dietary exposure and future proof against decreases to the regulatory limit. Finally, the developed method was successfully applied to analyze nine phthalates in eight commercially available vegetable oil.

## 1. Introduction

Phthalates are a class of chemicals that are used as plasticizers in various industries [1]. They can be found in a variety of products such as food contact materials, glues, electronics, personal-care products, medical equipment, tubing, packaging, children’s toys, etc. Some phthalates may be used in food packaging or other minor food contact uses such as components of adhesives, lubricants, and sealants. Because they are not chemically bound to products, leaching, migration, and evaporation during use can occur, resulting in human exposure and release into the environment. Phthalates may also be present in food due to migration from food contact materials, including processing equipment and packaging [2]. All phthalates have not been thoroughly studied, but there is evidence that some of them are harmful to our health [3]. Studies have shown that phthalates are associated with certain health problems, such as endocrine disruption, reproductive abnormalities, cancer, and low birth weight [4,5]. As a result, the use of certain phthalates is regulated in many countries. For example, on 7 July 2020, the restriction set out in Commission Regulation 2018/2005, on the placing on the market of articles that contain one or more of four phthalates, began to apply EU-wide [6]. In essence, the Regulation bans these phthalates in articles that cause exposure through the skin or by inhalation.

Since food is the major source of exposure to phthalates in humans, it is of great importance to assess toxicological levels of phthalates within it. Foodstuffs that are high in fat content, such as edible oil, are prone to phthalates contamination due to their lipophilicity, as well as direct exposure to food packaging and food contact materials [7,8]. Therefore, it is important to investigate the potential risks and dietary exposure of phthalates from edible oil since global consumption of edible oil is high. Tolerable Daily Intake (TDI) of some phthalates in food was proposed by the European Food Safety Authority (EFSA), even though the guideline is incomplete because of the absence of sufficient, reliable data. The TDI (mg/kg body weight/day) for dibutyl phthalate (DBP), benzyl butyl phthalate (BBP), diisononyl phthalate (DINP), diisodecyl phthalate (DIDP), and diethyl hexyl phthalate (DEHP) are 0.01, 0.5, 0.15, 0.15 and 0.05, respectively [9]. The European Union has set specific migration limits (SML) for phthalates of 0.3, 1.5, 9, and 30 mg/kg for DBP, BBP, DINP and DEHP, respectively [10]. In China, the GB 9685-2016 standard specifies the principles of use for additives in food contact materials and their products as well as the variety, use scope, maximum level, specific migration limit or maximum residue quantity, total specific migration limit and other limit requirements of their allowable use [11]. China has set SML of 1.5 and 0.3 mg/kg for DEHP and DBP in oily food [12]. Tang and co-workers considered a call for maximum permitted limits for phthalates in edible oil, considering the health risk and the need to facilitate international trade [13].

Suitable analytical methods are required to determine a range of phthalates at trace levels in complex food matrices to investigate dietary exposure and future proofs against decreases to the regulatory limits [14,15]. The analysis of phthalates in any food matrix is a challenge, not only because of the complexity of food, which may also contain other compounds that generate interferences or matrix effect, but also because they are ubiquitous in any analytical laboratory. Problems with analytical blanks can compromise sensitivity giving higher exposure estimates and possibly erroneous ‘false positive’ detections. Most analytical methods for the determination of phthalates in foodstuffs are based on gas chromatography coupled to mass spectrometry (GC–MS or GC-MS/MS), with electron ionization (EI). In one of the studies by Barp et al., a quantitative method was developed for ten phthalates in eight edible oil using solid-phase microextraction (SPME) combined with GC-MS/MS. A simple and rapid SPME method was used to minimize sample manipulation. The method was well validated with good linearity (coefficient of correlation > 0.996) and good repeatability for both intra- and inter-day assessment (coefficient of variation < 10%) [16]. Shi et al. developed a GC-MS method to simultaneously analyze five phthalates in 34 edible oil and 28 oilseed samples, with the aid of ultrasonic-assisted solvent extraction and solid-phase extraction (SPE) sample preparation. The recoveries of phthalates in both the edible oil (ranging from 72.4% to 103.0%) and oilseed (ranging from 77.2% to 98.8%) samples were excellent [17]. In another study by Lamb et al., the selectivity and sensitivity of phthalates were enhanced by using the selective ion monitoring (SIM) mode of a single quadrupole GC-MS. The recoveries of all 13 phthalates analyzed across three separate spiking levels at 5, 25, and 50 µg/kg were between 80% and 102% [18]. However, EI takes place at 70 eV and most phthalates are easily fragmented to generate the same ions, such as the dominant phthalic anhydride ion at *m/z* 149, whereby no molecular ions or only a small abundance of molecular ions can be observed in the mass spectra. Acquisition methods typically select the fragment ions (e.g., *m/z* 149, 163, 177) to detect and quantify all the phthalate analytes. The absence of any molecular ion makes it difficult to differentiate between the different phthalates, with identification reliant on chromatographic separation. This is particularly challenging when assessing data for di-isononyl phthalate (DINP) and di-isodecyl phthalate (DIDP), as GC analysis results in two overlapping clusters of unresolved peaks corresponding to different branched isomers. Monitoring less specific lower mass fragment ions also reduces the selectivity, which is especially important when analyzing complex sample matrices such as edible oil. Atmospheric Pressure Chemical Ionization (APCI) is a soft and universal ionization technique that can be used to produce radical cations and/or protonated molecules using nitrogen and corona discharge. The mechanism of the APCI technique is gas-phase ion-molecule reactions. Two main ionization pathways can occur: charge transfer or proton transfer. In charge transfer ionization, M^+●^ ions are formed, while in proton transfer ionization, [M+H]^+^ ions are formed. Mass spectra are typically dominated by molecular or protonated molecular ions and less fragmentation is observed, providing better sensitivity and selectivity enhanced if either the molecular/protonated ion or a high mass fragment ion is selected as the precursor ion for MRM transitions in MS/MS. APCI was first introduced for GC-MS analysis in the 1970s [19], but it was the introduction of the commercial APGC source for GC-MS/MS which prompted an expansion of applications for the determination of pesticide residues and various persistent organic pollutants, including dioxins [20,21,22,23].

Edible oil can be further subdivided into plant/vegetable oil, animal oil, or synthetic liquid fat, and we focused on vegetable oil in this study. The aim of this study was to develop and validate a simple sample preparation and quantification method for the simultaneous analysis of nine phthalates in eight vegetable oil using APGC-MS/MS. The nine phthalates of interest were dimethyl phthalate (DMP), diethyl phthalate (DEP), dipropyl phthalate (DPP), diisobutyl phthalate (DiBP), dibutyl phthalate (DBP), benzyl butyl phthalate (BBP), bis(4-methyl-2-pentyl) phthalate (BMPP), diethyl hexyl phthalate (DEHP), and dioctyl phthalate (DOP). Finally, this developed method was applied to the analysis of eight commercially available vegetable oil: palm oil, corn oil, coconut oil, canola oil, olive oil, and soybean oil.

## 2. Materials and Methods

### 2.1. Samples and Reagents

Analytical grade phthalates DMP, DEP, DPP, DiBP, DBP, BBP, BMPP, DEHP, and DOP were purchased from Sigma-Aldrich Corporation (St. Louis, MO, USA). A standard stock solution of nine phthalates was prepared in methanol at a concentration of 20 mg/L. Benzyl benzoate (purity > 99%) was used as the internal standard (IS) and purchased from Sigma-Aldrich Corporation. The benzyl benzoate solution was prepared in methanol at a concentration of 100 mg/L. LC-MS grade methanol and acetonitrile (ACN) were obtained from Fisher Scientific Corporation (Pittsburgh, PA, USA). Eight commercially available vegetable oil, palm oil, corn oil, coconut oil, canola oil, olive oil, and soybean oil were purchased from a local supermarket in Malaysia. The chemical compositions of the vegetable oil cited from the literature are provided in Supplementary Materials (Table S1) [24,25]. 

### 2.2. Sample Preparation

Cross-contamination from plastic materials is a common problem in phthalates analysis; thus, only glassware was used in this study for sample preparation. All the glassware was washed with detergent, followed by pure water (X 3), and dried under 320 °C for 2 h before use.

Phthalates-free refined palm oil was used as the sample matrix during method development. 0.5 g of palm oil was weighed in a 10 mL glass centrifuge tube. The phthalates calibration standard solutions were prepared at a serial concentration of 0.04, 0.1, 0.2, 0.5, 0.8, 1.0, 2.0, and 3.0 mg/kg by spiking the stock standard solution into the pre-weighed palm oil. Spiked samples were also prepared at a concentration of 0.2 and 1.0 mg/kg. Then, 5 µL of IS and 5 mL of ACN were added. The mixture was vortexed for 1 min using a vortex mixer and sonicated for 20 min in an ultrasound bath. The mixture was centrifuged at 5000 rpm for 5 min and 1 mL of the top layer was aliquoted out for analysis.

### 2.3. Phthalates Analysis by APGC-MS/MS

The analysis was done using an Agilent^®^ 7890B gas chromatograph (Agilent Technologies Inc., Santa Clara, CA, USA) coupled to a Waters^™^ Xevo^™^ TQ-S micro tandem quadrupole mass spectrometer (Waters Corporation, Milford, MA, USA), fitted with an APGC source, operated in positive ion mode, and controlled by MassLynx^™^ 4.2 software. A Restek^™^ Rxi^™^-5ms capillary column (30 m × 0.25 mm × 0.25 µm, Restek Corporation, Bellefonte, PA, USA) was used for GC separation. The oven temperature was set at 60 °C (2 min hold) initially, increased to 320 °C at 25 °C/min, and finally maintained at 320 °C for 5 min. Helium (99.999% purity) was used as a carrier gas in a constant flow of 1.2 mL/min and the injection volume was 1 µL with an autosampler in the split ratio of 1:10. The mass spectrometer parameters were set as follows: corona current at 0.4 µA, cone voltage at 0 V, cone gas (N_2_) at 20 L/h under wet condition and 90 L/h under dry condition, auxiliary gas (N_2_) at 250 L/h, and make-up gas (N_2_) at 275 mL/min. The initial instrument setup provides dry conditions, whereas wet conditions are obtained by adding water to the source region using a vial in a holding tray placed in the source enclosure. 

## 3. Results and Discussion

### 3.1. Optimizing APGC-MS/MS Conditions for Phthalates Analysis

**Figure 1:**
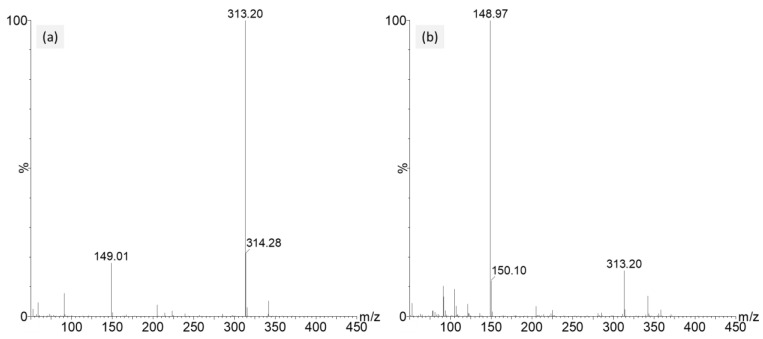
Mass spectra of BBP in full scan mode under wet (**a**) and dry (**b**) conditions.

There are two ionization mechanisms when using APGC: charge transfer and proton transfer. The initial step is the ionization of the nitrogen at the corona pin. For charge transfer, analyte molecules are directly ionized, resulting in radical cations. This form of ionization is generally favored by non-polar compounds. In many cases, protonation is directly produced by the traces of water present in the N_2_ supply. However, it can be enhanced using protic solvents as modifiers. Water molecules are first ionized, yielding oxonium ions (H_3_O^+^) that then ionize the analyte molecules via proton transfer resulting in protonated molecular ions ([M+H]^+^). This form of ionization is generally favored by relatively polar compounds. It is possible to promote either proton transfer or charge transfer in APGC by altering the source conditions and parameters such as cone gas. For proton transfer under wet source conditions, H_2_O should be drawn into the source by the auxiliary gas; thus, low cone gas flow values are used. On the contrary, charge transfer under dry source conditions is promoted using high cone gas flow values, as ideally no water enters the source. BBP was used as the representative phthalate and the mass spectra of the two different conditions are illustrated in Figure 1. Under wet conditions, proton transfer is the main mechanism, and the [M+H]^+^ ion at *m/z* 313 was observed as the major ion in the mass spectrum (Figure 1a). Charge transfer was investigated under dry conditions. Despite ultrahigh nitrogen quality with efficient nitrogen filters to trap residual water, the mass spectrum showed no presence of radical cations but was dominated by the phthalic anhydride fragment ion at *m/z* 149 and some evidence of the [M+H]^+^ ion at relatively low intensity (Figure 1b), attributed to uncontrolled traces of protic donors. The wet conditions were chosen for the MRM optimization. Low corona current and cone voltage were used to reduce potential in-source fragmentation and increase the intensity of the [M+H]^+^ ion. The corona current and cone voltage were determined as 0.4 µA and 0 V, respectively.

**Figure 2:**
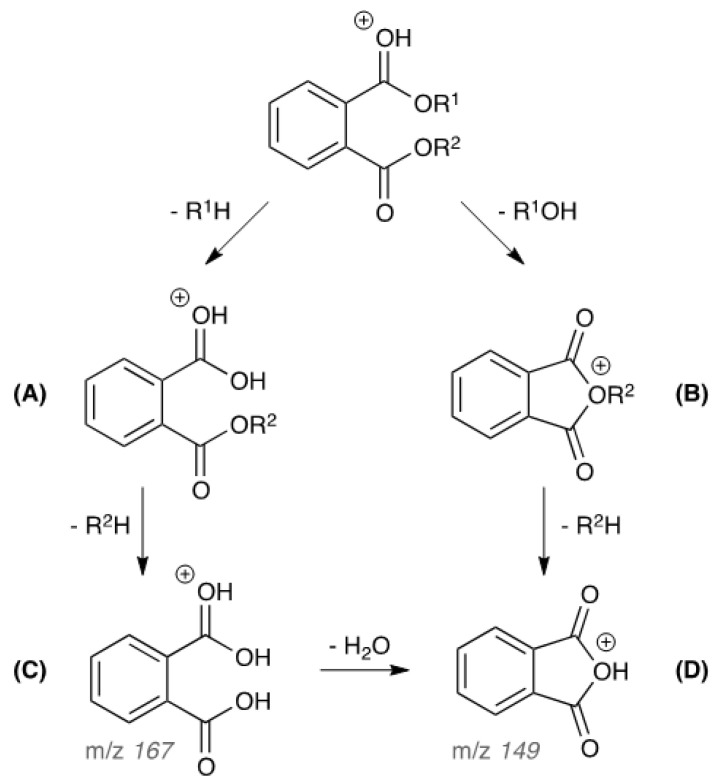
The proposed CID fragmentation pathway of phthalates by APGC-MS/MS under wet conditions.

The fragmentation pathway, produced by Collision Induced Dissociation (CID) of the tandem quadrupole mass spectrometer system, of phthalates by APGC-MS/MS under wet conditions was proposed in Figure 2. The phthalates produce fragment ion B by a loss of the corresponding neutral alcohol (R^1^OH) from the side chain, while some phthalates such as BMPP and DEHP can also produce fragment ion A by a loss of the corresponding alkene (R^1^-H) from the side chain. Generally, fragment ion B is the major product ion as compared to fragment ion A. Fragment ion B can be further converted to ion D (*m/z* 149) by the loss of a neutral alkene. The ion C (*m/z* 167) is a characteristic fragment ion observed from phthalates by consecutive loss of neutral alkene moieties. The ion C (*m/z* 167) can be further converted to ion D (*m/z* 149) through the loss of H_2_O.

The MS conditions for the analysis of nine phthalates in MRM mode were summarized in Table 1. All MRM transitions were established based on the selection of the [M+H]^+^ ions as the precursor ions. The product ion with the highest abundance was selected as the quantitation ion, and another product ion with the second highest abundance was chosen as the qualifier ion.

**Figure 3:**
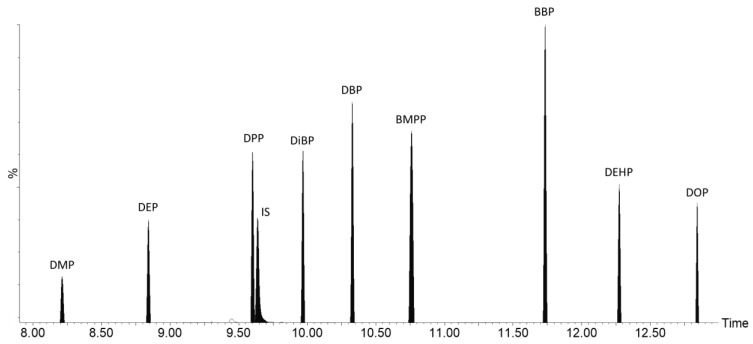
Chromatogram showing the main MRM transition for the nine phthalates and the IS.

The chromatogram of nine phthalates and the IS are shown in Figure 3. All the compounds were eluted within 14 min and well-resolved by both chromatographic separation and characteristic MRMs. 

### 3.2. Matrix Effect (ME) 

Matrix effects are a major concern in quantitative analysis because they can adversely affect the method’s accuracy, precision, and sensitivity [26]. Matrix effects can be produced by unwanted interactions in the GC injector. Analytes interact with active sites on the surface of the GC-liner and column to cause peak tailing, loss of response, and degradation of susceptible compounds. Matrix co-extractives, presence in excess, block active sites and protect analytes resulting in relative enhancement of response. As the ionization mechanism is APCI, the matrix effect may also be caused by changes in the ionization efficiency of target analytes in the presence of co-eluting compounds in the sample matrix. Palm oil was used as the representative sample matrix for vegetable oil in this study. The matrix effect was evaluated by comparing the analytes in the ACN solvent solution with analytes in the ACN-extracted matrix spiked with the same concentration of analytes. The matrix effect of the nine phthalates and IS were in the range of 18.4% to 27.4% calculated using Equation (1). The results showed that no significant matrix effects were observed when using ACN as the extraction solvent during sample preparation. Moreover, the use of benzyl benzoate as the IS can correct matrix effects while improving the method’s accuracy, precision, and recovery.

(1) $$\mathrm{ME}\;\left(\%\right)=\frac{\mathrm{peak}\;\mathrm{areas}\;\left(\mathrm{fortified}\;\mathrm{extract}\right)-\mathrm{peak}\;\mathrm{areas}\;\left(\mathrm{solvent}\right)}{\mathrm{peak}\;\mathrm{areas}\;\left(\mathrm{solvent}\right)}\times 100\%$$

### 3.3. Method Detection Limit (MDL) and Method Quantitation Limit (MQL)

Calibration curves at eight different concentration levels (4, 10, 20, 50, 80, 100, 200, and 300 µg/L) were built by spiking the IS and phthalates standard solution into the palm oil matrix. The values for the coefficient of determination (R^2^) were all >0.99, indicating that there is a good fit for all nine phthalate species. The method detection limit (MDL) and method quantitation limit (MQL) were established using the spiked concentration level of 4 µg/L. The MDL and MQL were calculated by multiplying the standard deviation of the nine replicates by 2.896 (Student’s t-value at 99% confidence level) and 10, correspondingly [27]. The calculated MDL and MQL in ACN solution were in the range of 0.43–1.67 and 1.50–5.80 µg/L, respectively (Table 2). According to the Wisconsin Department of Natural Resources, the inequality (calculated MDL < spiked concentration < 10 × calculated MDL) is used to evaluate a calculated MDL [28]. As these conditions are met, it is appropriate to use the spiked concentration of 4 µg/L to establish MDL and MQL. Additionally, DBP and DEHP are commonly detected in the environment, such as laboratory air, instrument, sample bottles and caps, and other sample processing hardware that can cause an elevated baseline in the chromatograms. According to US EPA method 556.1, the background interference should be less than half of the minimum reporting limit under the analysis condition [29]. Stringent quality control procedures were employed through the analytical protocol to reduce potential contamination to an acceptable level. We found that the intensities of DBP and DEHP at MQL were almost four times the process blank; thus, 4 µg/L can be regarded as a suitable concentration to establish the MDL and MQL for DBP and DEHP in this study. The corresponding concentration of phthalates in palm oil was calculated according to Equation (2).

(2) $$\;\;\;\;X=\rho \cdot \frac{v}{m}$$

X is the concentration in palm oil (mg/kg), v is the volume of solvent used in extraction (mL), m is the sample wight (g), and ρ is the concentration of phthalate calculated from calibration curve (µg/L). 

The MQL of all phthalates in palm oil was in the range of 0.015–0.058 mg/kg, which is much lower than the reported SML in China (i.e., 0.3 and 1.5 mg/kg for DBP and DEHP, respectively) [11]. Similar values were reported by Barp and Tang, but these methods were more complicated, using direct immersion solid-phase microextraction and solid-phase extraction (SPE), respectively, to concentrate and clean up extracts prior to injection onto GC-MS(/MS) [13,16]. Similar clean-up strategies and/or solvent exchange for allowing larger volume injection (i.e., from ACN to hexane) can be adopted here to further lower the detection limit if required. However, increasing the number of sample preparation steps has the tendency for cross-contamination, additional time hours, as well as increased consumables cost. In this study, we achieved good MQL and MDL with a simplified sample preparation process and a low injection volume. Acetonitrile has a large expansion volume, which limits the injection volume that can be used with conventional split/splitless injection and impacts sensitivity. Although this issue can be avoided by either using solvent exchange into another solvent such as toluene or by switching to a different design of injector, the programmable temperature vaporizer (PTV) with solvent vent, the capability to achieve sufficient sensitivity using a 1 µL injection of ACN, with an easy-to-use conventional split/splitless injection unit, is an attractive option.

### 3.4. Accuracy

The accuracy of the method was evaluated using 0.2 and 1 mg/kg of phthalates spiked in palm oil. The analyses were done using seven intra-day replicates and duplicates over a span of three days (Table 3). The recovery of nine phthalates at 0.2 and 1 mg/kg were in the range of 89–112% and 89–114%, respectively. The relative standard deviation (RSD) values for phthalates from the analysis at these two concentrations in both intra-day and inter-day conditions were <15%, with the majority < 10%, by calculating against the IS. The repeatability (intra-day) and reproducibility (inter-day) achieved in this study with a single internal standard were shown to be sufficient even without the use of individual stable isotope-labeled internal standards for each corresponding phthalate species. 

### 3.5. Analysis of Phthalates in Different Types of Vegetable Oil 

The established method was applied to analyze phthalates in eight different vegetable oil: palm oil, olive oil, canola oil, corn oil, and coconut oil. Among the nine phthalates sought, only DBP and DEHP were detected in some of the oil samples. DBP was detected in three out of the eight tested oil samples, and its concentration in palm oil 2, soybean cooking oil, and corn oil were 0.12 ± 0.02, 0.13 ± 0.02, and 0.06 ± 0.004 mg/kg, respectively. DEHP was detected in all oil samples, except coconut oil, and its concentration was in the range of 0.05–0.39 mg/kg (Table 4). In general, the concentration and occurrence of DEHP were found to be higher than DBP among the eight oil samples tested. The level of DBP and DEHP detected in the tested oil samples did not exceed the SML in China (e.g., 0.3 and 1.5 mg/kg for DBP and DEHP, respectively), and the results suggested that all the tested commercial vegetable oil from a local supermarket met the safety requirement of phthalates in edible oil.

## 4. Conclusions

In this study, a complete workflow consisting of a simple ACN extraction, and a quantitative analytical method based on APGC-MS/MS was developed to analyze nine phthalates in eight vegetable oil. This method has the following advantages, when compared to the other studies using GC-MS in EI mode. Firstly, the [M+H]^+^ ion was generated as the major precursor ion for each phthalate under wet conditions, which enhanced the selectivity and sensitivity during MRM optimization. This is especially important when handling complex sample matrices such as vegetable oil. Next, a simple sample preparation procedure with only ACN solvent extraction was required to achieve good accuracy and low MQL and MDL values with excellent linearity. The obtained MQL in vegetable oil was in the range of 0.015–0.058 mg/kg and is much lower than the SML. Finally, among the nine targeted phthalates, DBP was detected in three out of eight oil samples in the range of 0.06–0.13 mg/kg. DEHP was detected in seven oil samples in the range of 0.05–0.39 mg/kg. The level of the detected DBP and DEHP in the oil sample is below the recommended safety limit. Thus, this developed method can be applied to analyze phthalates in different matrices with minor modifications.

## Figures and Tables

**Table 1:** MS conditions for analysis of nine phthalates.

Compound	RT (Min)	Precursor Ion	Product Ion	Collision Energy
DMP	8.21	**195.0**	**163.0** *	7
DEP	8.84	223.1	149.0	15
		**223.1**	177.1 *	5
DPP	9.59	251.0	121.0	30
		**251.0**	**191.0** *	5
DiBP	9.96	279.0	121.0	40
		**279.0**	**205.0** *	5
DBP	10.32	279.0	121.0	40
		**279.0**	**205.0** *	5
BBP	11.73	313.2	91.0	20
		**313.2**	**205.0** *	5
BMPP	10.76	**335.0**	**167.0** *	10
		335.0	251.0	5
DEHP	12.27	391.0	149.0	10
		**391.0**	**167.0** *	10
DOP	12.84	391.4	149.0	20
		**391.4**	**261.2** *	10

* Transitions used for quantitation of phthalates.

**Table 2:** Linearity, method detection limit, and method quantitation limit in the solution and vegetable oil.

Compound	Linearity (R^2^)	MDL_sol_ (µg/L) ^a^	MQL_sol_ (µg/L) ^b^	MQL_oil_ (mg/kg) ^c^
DMP	0.998	0.71	2.45	0.025
DEP	0.997	1.67	5.75	0.058
DPP	0.999	0.94	3.24	0.032
DiBP	0.999	0.92	3.18	0.032
DBP	0.998	1.62	5.59	0.056
BBP	0.994	0.79	2.73	0.027
BMPP	0.997	0.43	1.48	0.015
DEHP	0.996	1.28	4.43	0.044
DOP	0.995	1.25	4.33	0.043

^a^ MDL_sol_ in ACN; ^b^ MQL_sol_ in ACN; ^c^ MQL_oil_ in palm oil.

**Table 3:** Recovery of 0.2 and 1 mg/kg phthalates in palm oil.

Compound	0.2 mg/kg				1 mg/kg			
	Intra-Day (*n* = 7)		Inter-Day (*n* = 6)		Intra-Day (*n* = 7)		Inter-Day (*n* = 6)	
	R * (%)	RSD (%)	R (%)	RSD (%)	R (%)	RSD (%)	R (%)	RSD (%)
DMP	104	4.41	105	8.66	106	4.82	98	4.56
DEP	99	12.0	101	7.05	114	3.67	109	4.76
DPP	112	3.54	103	8.19	113	2.92	106	5.29
DiBP	109	3.20	98	10.3	98	2.86	100	6.02
DBP	111	4.67	96	14.4	112	3.02	99	9.04
BBP	104	9.32	92	8.12	106	6.72	93	6.49
BMPP	108	7.28	95	8.92	107	3.72	94	7.96
DEHP	102	6.41	92	8.90	107	7.45	89	14.1
DOP	102	6.07	89	13.6	101	5.80	91	7.30

* R: recovery.

**Table 4:** Summary of phthalates in eight different vegetable oil (*n* = 3).

Oil Sample	DMP	DEP	DPP	DiBP	DBP	BBP	BMPP	DEHP	DnOP
Palm Oil 1	- ^a^	-	-	-	-	-	-	0.05 ± 0.004	-
Palm Oil 2	-	-	-	-	0.12 ± 0.02 ^b^	-	-	0.13 ± 0.02	-
Palm Oil 3	-	-	-	-	-	-	-	0.13 ± 0.02	-
Soybean Cooking Oil	-	-	-	-	0.13 ± 0.02	-	-	0.07 ± 0.01	-
Olive Oil	-	-	-	-	-	-	-	0.17 ± 0.03	-
Coconut Oil	-	-	-	-	-	-	-	-	-
Canola Oil	-	-	-	-	-	-	-	0.23 ± 0.04	-
Corn Oil	-	-	-	-	0.06 ± 0.004	-	-	0.39 ± 0.03	-

^a^ < MQL; ^b^ Unit: mg/kg.

## Data Availability

Not applicable.

## Supplementary Materials

The following supporting information can be downloaded at: https://www.mdpi.com/article/10.3390/toxics11030200/s1, Table S1: Some vegetable oil and their typical fatty acid composition (%).
