# Supplementary material for: Simultaneous Determination of Nine Phthalates in Vegetable Oil by Atmospheric Pressure Gas Chromatography with Tandem Mass Spectrometry (APGC-MS/MS)
Source: Toxics. 2023 Feb 21;11(3):200. doi: 10.3390/toxics11030200 (PMC10056964; doi:10.3390/toxics11030200)
Supplement: Supplementary file 1 [file toxics-11-00200-s001.zip › toxics-2202696-supplementary.pdf]

toxics

MDPI

Supplementary Materials: Simultaneous Determination of Nine Phthalates in Vegetable Oil by Atmospheric Pressure Gas Chromatography with Tandem Mass Spectrometry (APGC-MS/MS)

Yongjun Xiao, Wen Yee Wong, Li Yan Chan, Chee Keat Yong, Kosuke Abe, Peter Hancock and Simon Hird

Vegetable oil used in food consists of complex mixtures of triacylglycerols (TAGs, typically > 95%) with some small amounts of diacylglycerols (typically < 5%). Other minor components are tocopherols/tocotrienols (up to 900 mg kg^{-1}) and phytosterol esters/phytosterols (up to 1%) [1]. For different vegetable oil, the main components (TAGs) differ greatly in fatty acid composition (Table S1). They can be classified as short-chain (C6–8), medium-chain (C10–12), and long-chain (C14–18). The vegetable oil may be saturated (fully hydrogenated) or have one, two, or three double bonds [2].

Table S1. Vegetable oil and their typical fatty acid composition (%).

Oil	6:0	8:0	10:0	12:0	14:0	16:0	16:1	18:0	18:1	18:2	18:3	20:1	20:4	22:1
Palm oil				0.1	1.0	43.5	0.3	4.3	36.6	9.1	0.2	0.1		
Coconut	0.6	7.5	6.0	44.6	16.8	8.2		2.8	5.8	1.8				
Soybean					0.1	10.3	0.2	3.8	22.8	51.0	6.8	0.2		
Corn						10.9		1.8	24.2	58.0	0.7			
Olive						11.0	0.8	2.2	72.5	7.9	0.6	0.3		
Canola oil						4		2	55	26	10			2

References:

1. Hammond, E.W. VEGETABLE OILS/Types and Properties, in Encyclopedia of Food Sciences and Nutrition (Second Edition), 2003, 5899-5904. https://doi.org/10.1016/B0-12-227055-X/01225-6.

2. Dupont, J. VEGETABLE OILS/Dietary Importance, in Encyclopedia of Food Sciences and Nutrition (Second Edition), 2003, 5921-5925. https://doi.org/10.1016/B0-12-227055-X/01229-3.
